# Characteristics and clinical relevance of late gadolinium enhancement in cardiac magnetic resonance in patients with systemic sclerosis

**DOI:** 10.1007/s00380-014-0539-y

**Published:** 2014-07-05

**Authors:** Makoto Sano, Hiroshi Satoh, Kenichiro Suwa, Mamoru Nobuhara, Takeji Saitoh, Masao Saotome, Tsuyoshi Urushida, Hideki Katoh, Kumiko Shimoyama, Daisuke Suzuki, Noriyoshi Ogawa, Yasuo Takehara, Harumi Sakahara, Hideharu Hayashi

**Affiliations:** 1 Division of Cardiology, Internal Medicine III, Hamamatsu University School of Medicine, 1-20-1 Handayama, Higashi-ward, Hamamatsu, 431-3192 Japan; 2 Department of Emergency Medicine, Hamamatsu University School of Medicine, Hamamatsu, Japan; 3 Division of Immunology and Rheumatology, Internal Medicine III, Hamamatsu University School of Medicine, Hamamatsu, Japan; 4 Department of Radiology, Hamamatsu University School of Medicine, Hamamatsu, Japan

**Keywords:** Electrocardiography, Conduction disturbance, Cardiac function, Systemic scleroderma, Late gadolinium enhancement

## Abstract

Cardiac involvement in systemic sclerosis (SSc) is considerably frequent in autopsy, but the early identification is clinically difficult. Recent advantages in cardiac magnetic resonance (CMR) enabled to detect myocardial fibrotic scar as late gadolinium enhancement (LGE). We aimed to examine the prevalence and distribution of LGE in patients with SSc, and associate them with clinical features, electrocardiographic abnormalities and cardiac function. Forty patients with SSc (58 ± 14 years-old, 35 females, limited/diffuse 25/15, disease duration 106 ± 113 months) underwent serological tests, 12-lead electrocardiogram (ECG) and CMR. Seven patients (17.5 %) showed LGE in 26 segments of left ventricle (LV). LGE distributed mainly in the basal to mid inter-ventricular septum and the right ventricular (RV) insertion points, but involved all the myocardial regions. More patients with LGE showed NYHA functional class II and more (71 vs. 21 %, *p* < 0.05), bundle branch blocks (57 vs. 6 %, *p* < 0.05), LV ejection fraction (LVEF) < 50 % (72 vs. 6 %, *p* < 0.01), LV asynergy (43 vs. 0 %, *p* < 0.01) and RVEF < 40 % (100 vs. 39 %, *p* < 0.01). There was no difference in disease duration, disease types, or prevalence of positive autoimmune antibodies or high serum NT-proBNP level (>125 pg/ml). When cardiac involvement of SSc was defined as low LVEF, ECG abnormalities or high NT-proBNP, the sensitivity, specificity positive and negative predictive values of LGE were 36, 92, 71 and 72 %, respectively. We could clarify the prevalence and distribution of LGE in Japanese patients with SSc. The presence of LGE was associated with cardiac symptom, conduction disturbance and impaired LV/RV contraction.

## Introduction

Systemic sclerosis (SSc) is characterized by vascular changes and fibrosis of the skin and internal organs. The prevalence of cardiac involvement in SSc was considered clinically to be 1.4–5.4 % for impaired left ventricular ejection fraction (LVEF), or 18–30 % for diastolic dysfunction [1–3]. While in autopsy, myocardial fibrosis was identified in 50–80 % [4, 5]. In some pathological reports, cardiac involvement in SSc was assumed to be derived from impairment of the microcirculation and primary myocardial fibrosis, and from ischemic damage due to coronary atherosclerosis [6–8]. Patients with cardiac involvement have a poor prognosis because of the congestive heart failure and fatal arrhythmias associated with conduction disturbance [9].

Unfortunately, especially in the early phase, most patients with cardiac involvement are asymptomatic and difficult to be detected in subclinical stage. Previous studies have suggested that tissue Doppler echocardiography is useful for detection of the depressed contractility [3], and serum N-terminal-pro brain natriuretic peptide (NT-proBNP) can be a surrogate marker of cardiac involvement [10]. Recently, the values of cardiac magnetic resonance (CMR) are suggested for the early detection of cardiac involvement in SSc. Cine-CMR can assess cardiac morphology and function with high spatial resolution, and late gadolinium enhancement (LGE)-CMR can differentiate fibrotic scar from normal myocardium [11, 12]. Actually, in reports from Western countries, LGE was observed in 21–66 % of patients with SSc [13–15]. However, there are no such data in Asian patients, and only few studies have examined the values of LGE by comparison with echocardiographic findings and serum NT-proBNP level [13, 14].

This study aimed to assess the prevalence and distribution of LGE in patients with SSc, and to associate them with clinical features, electrocardiographical abnormalities and cardiac function.

## Patients and methods

### Patients

This was a single center trans-sectional study. We selected a total of 47 consecutive patients with SSc (17–77 years old) attending the division of immunology and rheumatology at Hamamatsu University Hospital between January 2012 and March 2013. The diagnosis of SSc was based on the guideline of Japanese Ministry of Health and Welfare [16], and the type of SSc was classified into limited or diffuse type according to the LeRoy’s classification [17]. Patients were excluded if they had (1) the history of coronary arterial disease, severe valvular diseases or obvious cardiomyopathies, (2) renal insufficiency with an estimated GFR <30 ml/min/1.73 m^2^, (3) implanted pacemaker, or (4) no informed consent. Finally, a total of 40 patients were enrolled in this study.

All patients underwent CMR, serological test and 12-lead electrocardiogram (ECG) within 1 month. The serological test included cardiac biomarkers such as NT-proBNP and troponin I, and autoimmune antibodies such as anti-Scl-70, anti-centromere and anti-U1-RNP. This study protocol was conducted in accordance with the Declaration of Helsinki and was approved by an institutional review board. All patients gave their informed consent.

### CMR protocol

CMR imaging was performed on a 1.5 tesla (T) MR system (Signa Infinity Twinspeed, GE Medical Systems, Waukesha, USA) with a gradient system performance of maximum amplitude of 40 mT/m and slew time of 150 T/m/s [18]. An 8-element phased array cardiac coil was used in all studies. Three planes such as short axis, sagittal long axis and 4-chamber view were obtained for 2-dimensional (2D) FIESTA cine images and LGE images. The slice thickness/gap was typically 10 mm/0 mm (6-9 slices). Breath-hold cine magnetic resonance images were obtained in contiguous short-axis planes from apex to base of the heart with the patient in a resting state. The 2D FIESTA cine images were based on the steady state free precession sequence. The imaging parameters were as follows; matrix of 192 × 192, field of view of 34 cm, flip angle of 45º, and readout bandwidth of 125 kHz. Sixteen data lines were acquired per each segment. The shortest repetition time and echo time were selected; however, the values were not exactly the same for each study, because they were related to the orientation of the scanning plane and slice thickness.

Late gadolinium enhancement images were acquired from 15 min after an injection of 0.2 mmol/kg of contrast material (Gd-DTPA-BMA, Fuji Pharma., Tokyo, Japan). LGE imaging was based on the inversion recovery prepared fast gradient echo (IR-FGRE) sequence. The imaging parameters were as follows; matrix of 256 × 160, field of view of 34 cm, flip angle of 20º, readout bandwidth of 31.25 kHz. The IR-FGRE technique repeated during every R-to-R interval and the trigger delay was 300 ms. The readout data line was 160 each, where 24 data lines were acquired per segment. The inversion time (200–240 ms) was individually determined right before the LGE imaging on basis to optimize nulling of the normal myocardium signal. The process to identify optimum contrast was concluded within 3 min.

### Analysis of CMR

Two experienced cardiovascular radiologists (M.S. and H.S.) interpreted all the CMR images without any knowledge of clinical findings. The 17-segments model was used for segmental analyses for morphology, function and LGE. LV/RV end-diastolic volume (LVEDV/RVEDV), end-systolic volume (LVESV/RVESV) and LV/RVEF were acquired from 2D FIESTA cine images in the short axis view. For LV/RV volume analysis, both the endocardial and epicardial contours for LV and only the endocardial contour for RV were manually traced in both end-diastole and end-systole phase, using analysis software (AW VolumeShare 2™, GE Medical Systems, Waukesha, USA). All the aortic, pulmonary and tricuspid valve rings were excluded from the volume. The LV and RV volume indices (LVEDVI/LVESVI and RVEDVI/RVESVI), and LV mass index (LVMI) were calculated by dividing them with body surface area. Asynergy in LV wall was also determined when several contiguous segments showed reduced contractility compared with other segments. LGE was defined as an area with a signal intensity which was higher than a signal intensity value >2SD above the normal myocardium, and was present in the same myocardial segment in at least two different planes. The presence, location, and pattern of myocardial LGE were determined by the consensus of the two observers. To assess LGE quantitatively, all the short-axis slices from base to apex were inspected visually, and in each image, the boundaries of LGE area were manually traced. The summed LGE area was rendered to LGE volume and the percentage against total muscle volume (%LGE volume) was calculated [18].

The pericardial involvement was also examined using cine- and LGE-CMR images. We evaluated the presence of pericardial effusion, and the grade of pericardial LGE as shown in constrictive pericarditis [19]. The presence of pericardial effusion and the grade of pericardial LGE were also determined by the consensus of the two observers.

### Statistical analyses

All the data were expressed as the mean ± standard deviation (SD) of the indicated numbers (*n*) or percentages, as appropriate. Categorical variables were compared between the patient groups by Chi-square or Fisher exact tests. Continuous variables between groups were examined by unpaired *t* test. Correlations between numerical parameters were evaluated by Pearson’s correlation. The differences were considered to be significant when *p* < 0.05. All the statistical analyses were performed using the software IBM SPSS (Ver.21).

## Results

### Prevalence and distribution of LGE

**Fig. 1:**
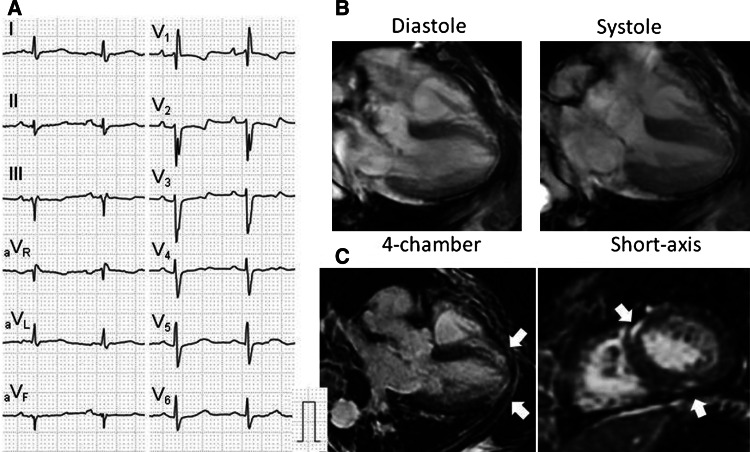
A representative case of SSc with LGE. Case 1 is a 67-year-old female patient who was NYHA class III with high NT-proBNP level (1,523 pg/ml). She had complete right bundle branch block with left axis deviation and non-specific ST-T abnormalities in 12-lead ECG (**a**). Cine-CMR showed normal LV volume and function (**b**). LGE-CMR exhibited striated and patchy types of LGE distributed in the mid-myocardium of anterior RV insertion point and inferior LV wall (**c**) (*arrows*)

**Fig. 2:**
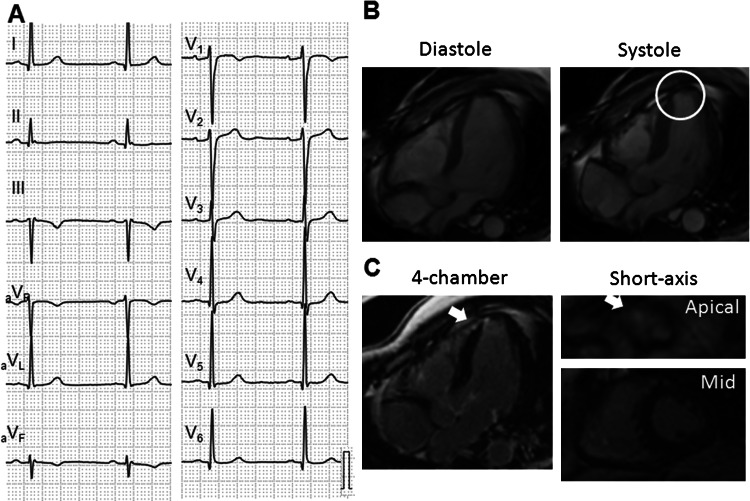
A representative case of SSc with LGE. Case 2 is a 47-year-old male who was asymptomatic and showed normal ECG, but had high NT-proBNP level (196 pg/ml) (**a**). Cine- and LGE-CMR showed asynergic wall motion and wall thinning (*circle*) with patchy LGE (*arrows*) in apical septum (**b** and **c**)

**Fig. 3:**
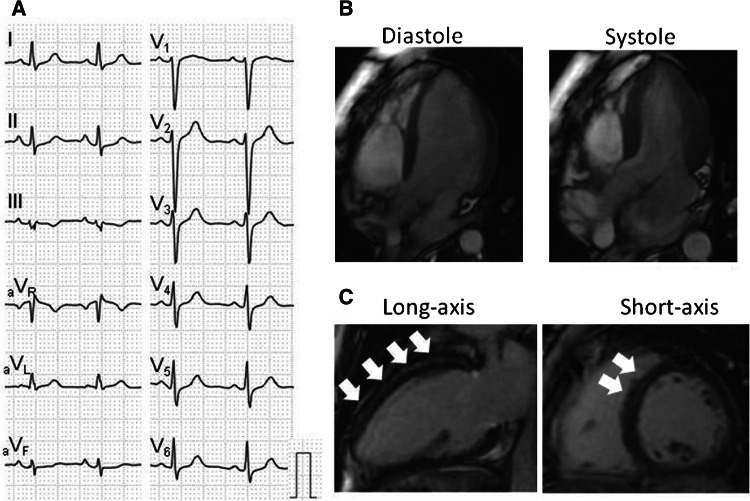
A representative case of SSc with LGE. Case 6 is a 31-year-old female patient who was asymptomatic and had normal NT-proBNP level (33 pg/ml) and normal ECG (**a**). Cine-CMR showed normal LV volume and function (**b**). LGE-CMR exhibited striated type of LGE distributed in the mid-myocardium of basal and mid LV wall (**c**) (*arrows*). The circumferential pericardial LGE was also apparent

We found 26 segments with LGE at the LV myocardium in 7 (17.5 %) patients with SSc. The representative three cases with myocardial LGE are shown in Figs. 1, 2 and 3. In general, LGE distributed mainly in the mid-myocardial wall of basal to mid-IVS but involved all myocardial regions. The intra-LV distribution of LGE was 11 IVS (including 1 anterior RV insertion point), 3 anterior, 6 lateral, 4 inferior, and 2 apical wall, respectively. The intra-mural distribution of LGE was 8 sub-epicardial, 13 mid-myocardial, and 5 sub-endocardial wall, respectively. The patterns of LGE were 23 striated and 3 patchy types. The mean %LGE volume was 2.7 % (range 0.7–7.9 %). The intra- and inter-observer variability for measurement of %LGE volume were acceptable (intra-observer *r* = 0.98, *p* < 0.001, inter-observer *r* = 0.98, *p* < 0.001). There was no significant correlation between %LGE volume and LVEF, RVEF, LVEDVI or RVEDVI (data not shown). No LGE was found in RV or atrial wall.

### LGE and clinical features

Table 1 shows general and immunological features in patients with SSc. There was no difference between patients with and without LGE in terms of age, sex, disease duration, disease type, prevalence of systemic hypertension, interstitial pneumonia, renal dysfunction or autoimmune antibodies. In overlaps of other autoimmune diseases, patients with LGE had 1 polymyositis (PM) and 1 chronic thyroiditis (CT), while patients without LGE had 3 systemic lupus erythematosus (SLE), 4 PM, 1 rheumatoid arthritis (RA), 1 polyarteritis nodosa (PN), and 4 CT. Medications were mainly corticosteroids, immunosuppressors and prostanoids. Calcium blockers, angiotensin-converting enzyme inhibitor and angiotensin II receptor blockers were not widely administered. The prevalence of other autoimmune diseases and medications did not differ between patients with and without LGE.

**Table 1:** General and immunological features in patients with and without LGE

	LGE (+)	LGE (−)	*p* value
Number	7	33	
Age (y.o.)	54.0 ± 22.8	59.3 ± 11.0	ns.
Female (%)	6 (86 %)	29 (88 %)	ns.
Disease duration (months)	49.7 ± 38.2	118.3 ± 120.5	ns.
Disease type (limited/diffuse)	3/4	11/22	ns.
Systemic hypertension	1 (14 %)	10 (30 %)	ns.
Interstitial pneumonia	3 (43 %)	15 (45 %)	ns.
30 ≤ eGFR < 60 ml/min/1.73 m^2^	0 (0 %)	6 (18 %)	ns.
Autoimmune antibodies			
Anti-Scl-70 antibody	1 (14 %)	4 (12 %)	ns.
Anti-centromere antibody	2 (29 %)	10 (30 %)	ns.
Anti-U1-RNP antibody	1 (14 %)	8 (24 %)	ns.
Overlaps			
SLE/PM/RA/PN/CT	0/1/0/0/1	3/4/1/1/4	ns.
Medications			ns.
Corticosteroids	4 (57 %)	12 (36 %)	ns.
Immunosupressors	1 (14 %)	6 (18 %)	ns.
Prostanoids	4 (57 %)	11 (33 %)	ns.
Calcium blockers	1 (14 %)	4 (12 %)	ns.
ACEI/ARBs	0 (0 %)	10 (30 %)	ns.

The categorical variables were expressed as number and percentage (%) and compared by Chi square test. The continuous variables were expressed as mean ± SD and examined by unpaired *t* test

*ACEI* angiotensin converting enzyme inhibitors, *ARB* angiotensin receptor blockers, *CT* chronic thyroiditis, *eGFR* estimated glomerular filtration rate, *PM* polymyositis, *PN* polyarteritis nodosa, *RA* rheumatoid arthritis, *SLE* systemic lupus erythematosus, *ns* not significant

Table 2 demonstrates cardiac features in all patients. Five patients (71 %) with LGE had New York Heart Association (NYHA) classes ≥ II, whereas nine (27 %) without LGE did (*p* < 0.05). Four patients with LGE showed high serum levels of NT-proBNP (>125 pg/ml), but seven without LGE also had, and the difference did not reach significant (57 vs. 21 %, *p* = ns.). In 12-lead ECG, 4 patients (57 %) with LGE showed bundle branch blocks (see Fig. 1), whereas only three patients without LGE (9 %) did (*p* < 0.05). Only one patient with LGE showed paroxysmal atrial fibrillation (Case 7). Three patients with LGE had normal ECG (see Figs. 2, 3).

**Table 2:** Cardiac features in all patients

Case no.	Age	Sex	NYHA class	NT-proBNP (pg/ml)	ECG abnormalities	PQ (ms)	QRS (ms)	QTc (ms)	LVEDVI (ml/m^2^)	LVEF (%)	RVEDVI (ml/m^2^)	RVEF (%)	LV asynergy	Pericardial effusion	Pericardial LGE
LGE (+)															
1	67	F	III	1,523	RBBB + LAH	150	96	469	45.1	75.9	62.6	31.0	–	–	–
2	47	M	I	196	N	170	104	402	68.9	47.7	80.0	20.0	+	–	Moderate
3	17	F	II	29	N	162	78	436	77.0	46.3	71.2	17.0	–	–	Moderate
4	71	F	IV	1,624	PAF, AVB, LBBB	294	148	538	152.3	24.8	89.5	24.0	+	+	Moderate
5	77	F	II	42	LAH	168	80	453	69.1	46.4	45.0	32.0	–	+	–
6	31	F	I	33	N	132	96	418	69.3	58.7	52.0	28.0	–	–	Severe
7	68	F	III	9,506	RBBB + LPH	–	144	440	95.2	33.7	123.8	21.0	+	+	–
LGE (−)															
8	67	F	II	40	N	128	82	441	46.3	70.4	55.0	48.0	–	+	–
9	56	M	II	91	N	154	98	428	63.3	54.5	71.7	54.0	–	–	Moderate
10	49	F	I	26	LAH	156	96	456	62.2	63.8	46.6	44.0	–	–	Mild
11	69	F	II	776	AVB, CRBBB, Q	–	134	481	91.8	44.2	81.1	25.0	–	–	Moderate
12	49	F	I	43	N	160	100	429	73.9	63.5	57.5	47.0	–	–	Mild
13	68	F	II	729	N	154	76	441	88.5	54.9	82.5	52.0	–	–	Moderate
14	70	F	I	103	N	130	78	398	37.4	73.6	32.9	56.0	–	–	Moderate
15	42	F	I	116	N	136	74	442	61.0	55.3	84.5	41.0	–	–	Moderate
16	54	F	II	308	N	168	82	450	61.7	49.2	63.7	30.0	–	–	Mild
17	56	F	II	92	N	178	86	414	68.0	52.3	57.3	30.0	–	–	Mild
18	65	F	I	156	N	156	90	427	85.6	64.6	54.9	44.0	–	+	–
19	65	F	I	95	N	130	84	441	54.0	74.3	61.3	63.0	–	–	Moderate
20	48	F	I	107	AVB	212	94	422	59.5	64.6	58.5	47.0	–	–	–
21	59	F	I	46	RBBB	176	142	422	74.0	62.7	108.1	36.0	–	–	Moderate
22	71	F	I	51	AVB	210	104	425	57.7	67.7	55.5	51.0	–	–	Mild
23	68	F	I	98	N	130	78	455	75.2	70.3	64.9	39.0	–	+	Moderate
24	71	M	II	70	N	142	108	438	64.3	59.1	64.6	28.0	–	–	Mild
25	52	F	I	38	N	120	74	415	54.3	65.8	36.0	31.0	–	–	–
26	62	M	I	5	N	136	88	409	72.4	56.9	83.7	37.0	–	–	–
27	60	M	I	42	N	172	72	426	67.2	55.1	67.3	31.0	–	–	Moderate
28	70	F	I	62	N	130	82	443	67.1	61.0	66.0	29.0	–	–	Mild
29	66	F	I	132	N	156	76	434	43.8	71.0	37.3	45.0	–	–	Mild
30	52	F	I	131	N	142	82	441	60.5	59.3	69.9	53.0	–	–	–
31	62	F	II	151	N	164	80	409	62.0	73.9	54.1	58.0	–	+	Moderate
32	77	F	I	82	N	186	82	404	52.7	76.3	49.9	45.0	–	–	–
33	57	F	I	99	N	184	98	409	46.9	68.5	57.8	39.0	–	–	–
34	57	F	II	84	N	170	80	438	42.7	79.9	62.1	49.0	–	–	–
35	20	F	I	42	N	118	90	429	59.3	65.2	55.6	38.0	–	–	Moderate
36	65	F	I	90	N	154	80	392	68.4	70.8	80.1	65.0	–	–	–
37	47	F	I	57	N	164	90	370	50.6	61.1	80.8	35.0	–	–	Mild
38	55	F	I	90	N	144	84	417	72.0	68.7	67.4	41.0	–	–	Moderate
39	61	F	I	63	N	154	88	413	49.9	82.1	55.7	56.0	–	–	Moderate
40	66	F	I	107	N	118	88	429	64.4	70.0	59.2	32.0	–	–	Mild

*AVB* atrio-ventricular block, *LAH* and *LPH* left anterior and posterior hemiblocks, *LBBB* and *RBBB* left and right bundle branch blocks, *LVEDVI* and *RVEDVI* left and right ventricular end-diastolic volume, *LVEF* and *RVEF* left and right ventricular ejection fractions, *NYHA* New York Heart Association, *PAF* paroxysmal atrial fibrillation, *Q* abnormal Q waves

**Fig. 4:**
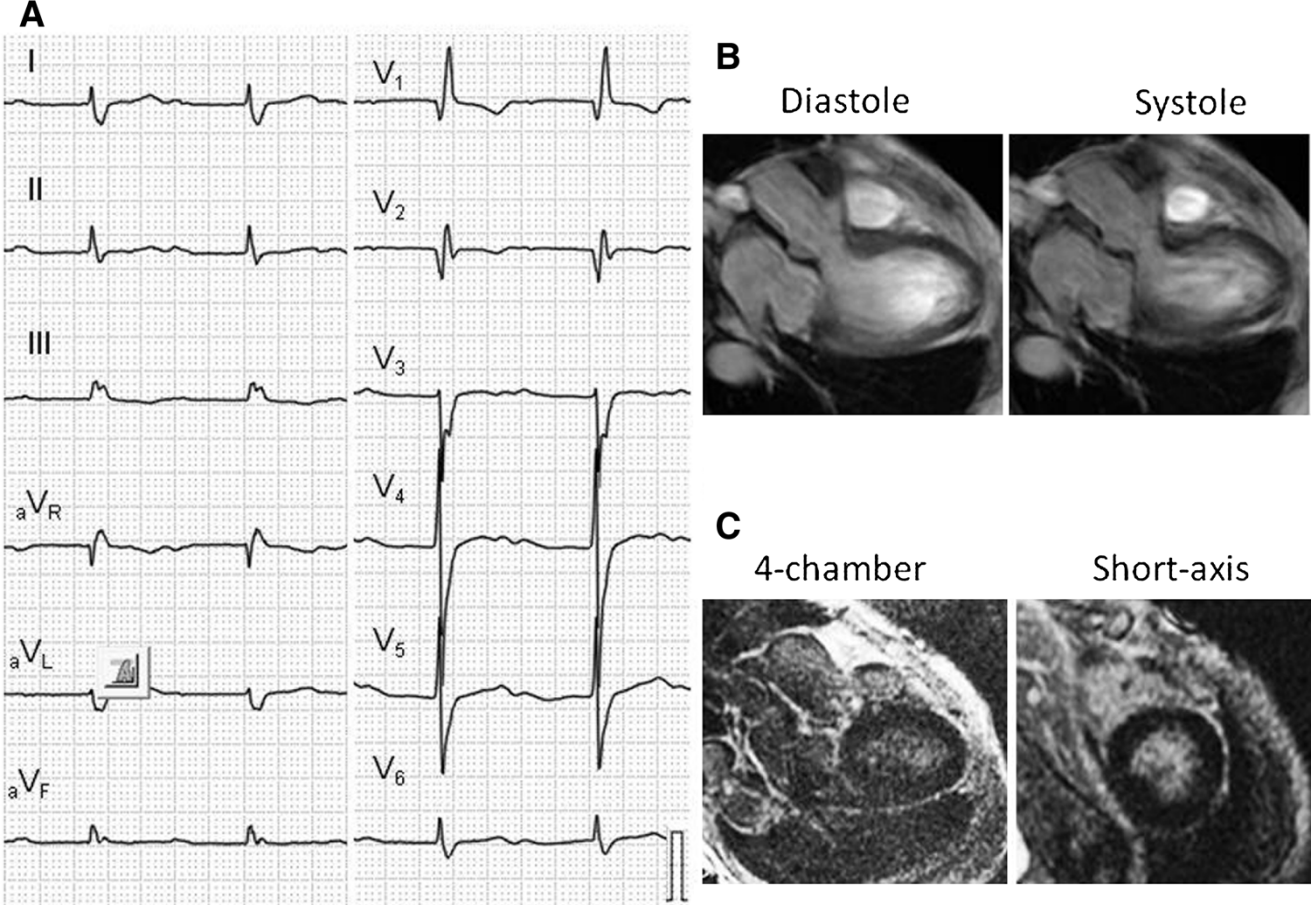
A representative case of SSc without LGE. Case 11 is a 69-year-old female who was NYHA class II with high NT-proBNP level (776 pg/ml). She had first degree atrio-ventricular block, right bundle branch block and abnormal Q waves (**a**). Cine-CMR showed LV dilatation and globally impaired LV contraction **(**LVEF = 44 %, (**b**), but LGE-CMR demonstrated no LGE in myocardium (**c**). The circumferential pericardial LGE was also apparent

In cine-CMR, five patients with LGE showed low LVEF (<50 %) and three of them had asynergic wall motion in segments with LGE (*p* < 0.05, see Fig. 2). However, two patients without LGE also showed low LVEF. Additionally, all patients with LGE showed RVEF < 40 %, whereas 13 patients (39 %) without LGE did. Case 11 (Fig. 4) is a 69-year-old female who was NYHA class II with high NT-proBNP level. She had first degree atrio-ventricular block, right bundle branch block and abnormal Q waves. She also showed LV dilatation and globally impaired LV contraction, but had no LGE in myocardium.

The pericardial effusion was observed in three patients (43 %) with LGE and four (12 %) without LGE. Mild to severe pericardial LGE was seen in four patients (57 %) with LGE and 23 (70 %) without LGE. No patient demonstrated pericardial thickness over 4 mm. The prevalence of pericardial effusion or LGE did not differ between patients with and without LGE (86 vs. 76 %, *p* = ns.).

### Diagnostic values of LGE for cardiac involvement of SSc

When cardiac involvement of SSc was defined as low LVEF, ECG abnormalities or high NT-proBNP (except patients with eGFR <60 ml/min/1.73 m^2^), the sensitivity and specificity of LGE were 36 and 92 %, and positive and negative predictive values were 71 and 72 %, respectively.

## Discussion

This study examined CMR in patients with SSc and showed that (1) LGE in myocardium was considerably frequent, (2) LGE distributed mainly in the basal to mid IVS and the RV insertion points, but involved all myocardial regions, and (3) more patients with LGE were symptomatic and had ECG abnormalities, low LV/RV EF and asynergy. We could clarify, for the first time, the prevalence and distribution of LGE, and the association with ECG abnormalities and impaired cardiac function in patients with SSc.

### Myocardial fibrosis and LGE in SSc

In autopsy, myocardial fibrosis in SSc was identified in 50–80 % [4, 5]. Bulkley et al. [5] reported that myocardial lesions in SSc consisted of contraction band necrosis, focal fibrotic changes, and muscle cell necrosis, and replacement fibrosis. The focal fibrosis was randomly localized throughout the layers of myocardium both in RV and LV. In endomyocardial biopsy, patients with SSc had more interstitial collagen volume fraction than normal controls regardless of signs of heart failure [20]. However, the endomyocardial biopsy is an invasive manner, and it has been in demand for non-invasive evaluation of fibrotic lesion.

This study showed that LGE has been distributed in 7 (17.5 %) of 40 Japanese patients with SSc. In previous reports from Western countries, LGE was observed in 21–66 % [13–15]. The difference in the prevalence of LGE might result from variations in the patient populations including racial differences.

### Mechanisms of fibrosis and LGE in SSc

In pathological studies, myocardial fibrosis in SSc was assumed to be derived from impairment of microcirculation due to micro-vascular abnormalities or primary myocardial fibrosis, and from ischemic damage due to coronary atherosclerosis [6–8]. In addition, mononuclear cell infiltration, mainly consisted of CD-3 positive T cells, has been reported in myocardial biopsies [7, 21]. In our findings, LGE distributed mainly in the basal to mid-IVS, but involved all myocardial layers. Although LGE regions involved sub-endocardial layers, the patchy types of distribution and discordance with certain coronary perfusion areas might exclude the myocardial infarction due to coronary atherosclerosis. Tzelepis et al. [13] also reported that all regions with LGE were localized in the basal segments and exhibited linear pattern in the mid-myocardial layer with spared sub-endocardium. Hence, LGE in SSc is likely to be caused by various etiologies including dilated cardiomyopathy (DCM)-like mid-wall fibrosis, inflammation, and ischemia [22, 23]. The additional analysis with T2-weighted CMR may help to clarify the intimate mechanism of LGE in patients with SSc [24].

However, although LGE-CMR can differentiate the myocardial fibrotic scar from normal myocardium [11], diffuse fibrosis cannot be visualized, because myocardium with diffuse fibrosis was “nulled” to highlight focal scar [25]. In the present study, a small number of patients without LGE showed low LVEF, suggesting undiagnosed LV wall damage in such patients.

Additionally, several studies have shown that patients with pulmonary arterial hypertension have LGE in the RV insertion points and in IVS [26], and that the presence of LGE correlates with RV dysfunction and poor prognosis [27, 28]. In this study, two patients had LGE in the RV insertion points, and all patients with LGE exhibited low RVEF [29]. Hesselstrand et al. [30] reported that LGE in the RV insertion points is a characteristic feature of connective tissue disease-related pulmonary arterial hypertension, although the mechanism is unknown.

### Clinical relevance of LGE in SSc

Many studies have associated LGE with clinical and ECG features and long-term cardiac events in patients with ischemic and non-ischemic cardiomyopathies [22], [31–36]. Here we examined whether LGE might be a surrogate marker of cardiac involvement in SSc. We showed that more patients with LGE were symptomatic (NYHA classes ≥ II), although there was no difference in the prevalence of high serum NT-proBNP level. The patients without LGE included those with low eGFR might affect the negative result (see Table 1). Allanore et al. [10] reported that serum NT-proBNP level can be a surrogate marker of cardiac involvement in SSc.

The presence of LGE was not associated with disease duration, disease types, or autoimmune antibodies. However, Tzelepis et al. [13] showed a close relationship between LGE volume and the duration of Raynaud’s phenomenon, and Steen et al. [37, 38] reported significant correlations of anti-Scl 70 and anti-centromere antibodies with organ involvements.

In 12-lead ECG, more patients with LGE had bundle branch blocks. Follansbee et al. [39] reported that in 102 patients with SSc, ventricular conduction abnormalities were present in 17 %. The finding that LGE was mainly localized in IVS might be associated with higher prevalence of conduction disturbance.

In cardiac function, previous reports showed that even asymptomatic patients with SSc had systolic and diastolic dysfunction in echocardiography [40–42]. In this study, more patients with LGE had low LVEF, LV asynergy, and low RVEF. However, there were no significant correlations between %LGE volume and LV/RV EDVI and EF. The reason was uncertain but a limitation of LGE-CMR in terms of estimation of diffuse fibrosis might cause the negative results. Furthermore, two patients with LGE showed normal NT-proBNP level, ECG, and LVEF. Thus, the ability of LGE-CMR to detect cardiac fibrosis in the subclinical stage may help identification of high risk patients and early initiation of therapeutic interventions, although the relevance in long term prognosis remains to be elusive.

Finally, the pericardial involvement was frequent in our patients with SSc, although the prevalence did not differ between patients with and without myocardial LGE. Previous necropsy studies showed pericardial diseases in 33–77 % of the cases, whereas any symptoms occurred in only 7–20 %. The pericardial involvement included fibrinous pericarditis, chronic fibrous pericarditis, pericardial adhesions, and pericardial effusions [5, 43]. We can also show the usefulness of pericardial imaging with CMR for the detection of pericardial involvement of SSc in asymptomatic patients.

### Limitations

First, as mentioned above, LGE-CMR cannot visualize diffuse fibrosis, because myocardium with diffuse fibrosis is regarded as normal by the nulling method. Actually, the sensitivity of LGE was low for cardiac involvement of SSc defined with other diagnostic modalities. However, previous studies and our data suggest the analysis of fibrotic scar with LGE-CMR still has clinical relevance [22, 33, 34]. This disadvantage might be referred to DCM, although novel T1 mapping techniques can quantitatively assess myocardial fibrosis [25]. Second, coronary angiography and endomyocardial biopsy were not routinely performed to exclude coronary arterial disease and idiopathic/secondary cardiomyopathies. However, both examinations are invasive and the use of contrast media should be avoided especially in patients with reduced renal function. Finally, small sample size and number of patients with LGE and the lack of prognostic evaluation might cause negative results for some clinical comparisons, and be limitations for extrapolating our data to diverse groups of patients. We did not compare the continuous variables for cardiac features, but just examined the prevalence of abnormal findings.

## Conclusions

This study could clarify the prevalence and distribution of LGE in patients with SSc. The presence of LGE was associated with cardiac symptom, conduction disturbance and impaired LV and RV contraction. Further studies are necessary to elucidate the relevance of LGE for early detection of cardiac involvement and for prediction of long term outcomes in patients with SSc.
